# Phage-quinolone synergy: mechanisms, clinical strategies, and translational prospects

**DOI:** 10.3389/fmicb.2026.1846582

**Published:** 2026-05-20

**Authors:** Xinrong Li, Shangsheng Yao, Guangtao Huang

**Affiliations:** Department of Burn and Plastic Surgery, Medical Innovation Technology Transformation Center, Shenzhen Second People's Hospital, The First Affiliated Hospital of Shenzhen University, Shenzhen, China

**Keywords:** antibiotic resistance reversal, antimicrobial resistance, bacterial biofilm, clinical administration strategies, mechanism of action, multidrug-resistant bacteria

## Abstract

The escalating global crisis of bacterial antimicrobial resistance (AMR) has significantly limited the clinical efficacy of quinolone antibiotics, while the use of phages alone suffers from inherent limitations, including a narrow host spectrum and the propensity to rapidly induce phage resistance. Consequently, combined antimicrobial therapy, defined as phage-antibiotic synergy (PAS), has emerged as a pivotal direction to address the challenges. The specific objectives of this review are to systematically dissect the bidirectional synergistic and antagonistic interactions between quinolone antibiotics and bacteriophages, define the key regulatory factors governing their combined effects, and establish an evidence-based framework to guide the standardized clinical application of quinolone-based PAS. This paper systematically reviews the mechanisms underlying the synergistic and antagonistic interactions between quinolone antibiotics and bacteriophages. The synergistic effects are manifested across multiple dimensions: bacterial morphological remodeling, activation of temperate phages, disruption of biofilm barriers, potentiation of functional proteins, dual regulation of resistance evolution, and synergy with the host immune system. In contrast, antagonistic effects are primarily triggered by high-concentration antibiotics interfering with phage proliferation, adaptive phenotypic alterations of bacteria, and imbalanced administration strategies. Simultaneously, we summarize standardized clinical optimization strategies, including sequential administration, sub-inhibitory antibiotic concentrations paired with the minimum effective multiplicity of infection (MOI) of phages, rational selection of broad-host-range and highly lytic phages, and individual regimen tailoring to match the specific infectious scenario and host immune status. We further analyze key challenges in the clinical translation of this therapeutic approach and propose future research directions, offering a framework to guide both mechanistic studies and clinical implementation of PAS as a strategy to mitigate antimicrobial resistance.

## 1 Introduction

Quinolones are among the most widely used broad-spectrum antibacterial agents in clinical practice worldwide. The first quinolone agent, nalidixic acid, was serendipitously discovered by researchers in 1962 during the development of antimalarial drugs, and received marketing approval from the U. S. Food and Drug Administration (FDA) in 1964. Through four generations of structural modifications and iterative optimization, the antibacterial spectrum of quinolones has been gradually expanded from exclusive coverage of Gram-negative (G^−^) bacteria to include Gram-positive (G^+^) bacteria and anaerobic bacteria, establishing them as first-line agents for the treatment of infections across diverse clinical scenarios (Jia and Zhao, 2021; Kaur et al., 2022). These agents exert their bactericidal effects by inhibiting bacterial DNA gyrase (topoisomerase II) and topoisomerase IV, thereby blocking bacterial DNA replication. With the advantages of excellent oral bioavailability, strong tissue penetration, and convenient administration (Patel and Goldman, 2016), they rank among the most prescribed antibacterial drug classes globally (Henry, 2017).

However, with the inappropriate use of quinolones in clinical settings and animal husbandry (Sproston et al., 2018), quinolone resistance has emerged since the early stage of their clinical introduction and continued to worsen over time. The underlying resistance mechanisms have evolved from initial chromosome-mediated mutations in the quinolone resistance-determining region (QRDR) of the target enzyme gene *gyrA*, to the horizontal transmission of plasmid-mediated quinolone resistance (PMQR) genes, which has accelerated the global pandemic of quinolone resistance. At present, quinolone resistance has become a major global public health challenge. The resistance rate to quinolones among multidrug-resistant (MDR) bacteria, represented by the ESKAPE pathogens, continues to rise (Miller and Arias, 2024), with frequent emergence of high-level and extensively drug-resistant (XDR) phenotypes. These relevant pathogens have been listed as the highest priority for the research and development of novel antibiotics by the World Health Organization (WHO) (Tacconelli et al., 2018).

The continuous deterioration of quinolone resistance has posed a severe clinical crisis: it not only directly increases the failure rate of anti-infective treatment, mortality of infected patients, and medical burden, but also, coupled with multiple black box warnings issued by the FDA for adverse drug reactions, further narrows the clinical application scenarios of quinolones, resulting in an extreme shortage of treatment options for MDR bacterial infections (Rodrigues and Silva, 2025). Meanwhile, although phage monotherapy has emerged as a promising novel therapeutic strategy with the advantages of targeted bactericidal activity and retained potency against drug-resistant bacteria, it still faces inherent limitations, including a narrow host range, the propensity to rapidly induce phage resistance, and limited efficacy against biofilm-associated infections.

The increasing prevalence of quinolone resistance (GBD 2021 Antimicrobial Resistance Collaborators, 2024), together with the limitations of antibiotic or phage monotherapy, has spurred interest in combination strategies that harness synergistic interactions between antibiotics and phages (Paranos et al., 2025b). Among these, phage–antibiotic synergy (PAS), particularly the combination of phages with quinolone antibiotics, represents a promising approach to address the therapeutic challenges posed by MDR bacterial infections (AlJerf et al., 2026). This combination exerts enhanced bactericidal effects through diverse mechanisms, including bacterial morphological remodeling, activation of temperate phages, biofilm disruption, potentiation of functional proteins, reciprocal modulation of resistance evolution, and cooperation with host immunity, while also delaying the emergence and spread of antimicrobial resistance (Supina and Dennis, 2025). However, under certain conditions, such as high antibiotic concentrations, specific bacterial strain characteristics, or imbalanced administration regimens, antagonistic interactions may occur (Tagliaferri et al., 2019). The net outcome of these interactions is shaped by multiple factors, including drug concentration, phage properties, bacterial traits, and microenvironmental conditions (Fatima and Hynes, 2025b).

Despite rapid progress in PAS research, existing studies have largely focused on individual mechanisms or specific bacterial species (Maad et al., 2026). A systematic synthesis that integrates the multifaceted mechanisms, key regulatory factors, optimization strategies, and translational pathways of quinolone-based PAS remains lacking (Moghadam et al., 2025). In this review, we provide a comprehensive overview of the core synergistic and antagonistic mechanisms underlying phage–quinolone interactions, analyze the key factors that govern their combined effects, and discuss strategies for clinical optimization as well as existing translational challenges. By establishing an integrated framework that spans molecular mechanisms to clinical application, we aim to offer researchers a clear perspective on the current landscape and future directions in this field, while providing clinicians with practical guidance for the standardized use of quinolone-based PAS.

## 2 Quinolone antibiotics and their resistance mechanisms

Quinolone antibiotics are a class of broad-spectrum antibacterial agents (Hooper and Jacoby, 2016; Yang et al., 2018). They inhibit bacterial proliferation and induce cell death by targeting bacterial DNA gyrase and topoisomerase IV, thereby blocking the formation of DNA supercoiling and the replication process (Shariati et al., 2022). However, the increasing emergence of drug-resistant strains has severely compromised their clinical efficacy, rendering in-depth investigation of resistance mechanisms and exploration of combination therapeutic strategies of great clinical and scientific significance.

**Figure 1:**
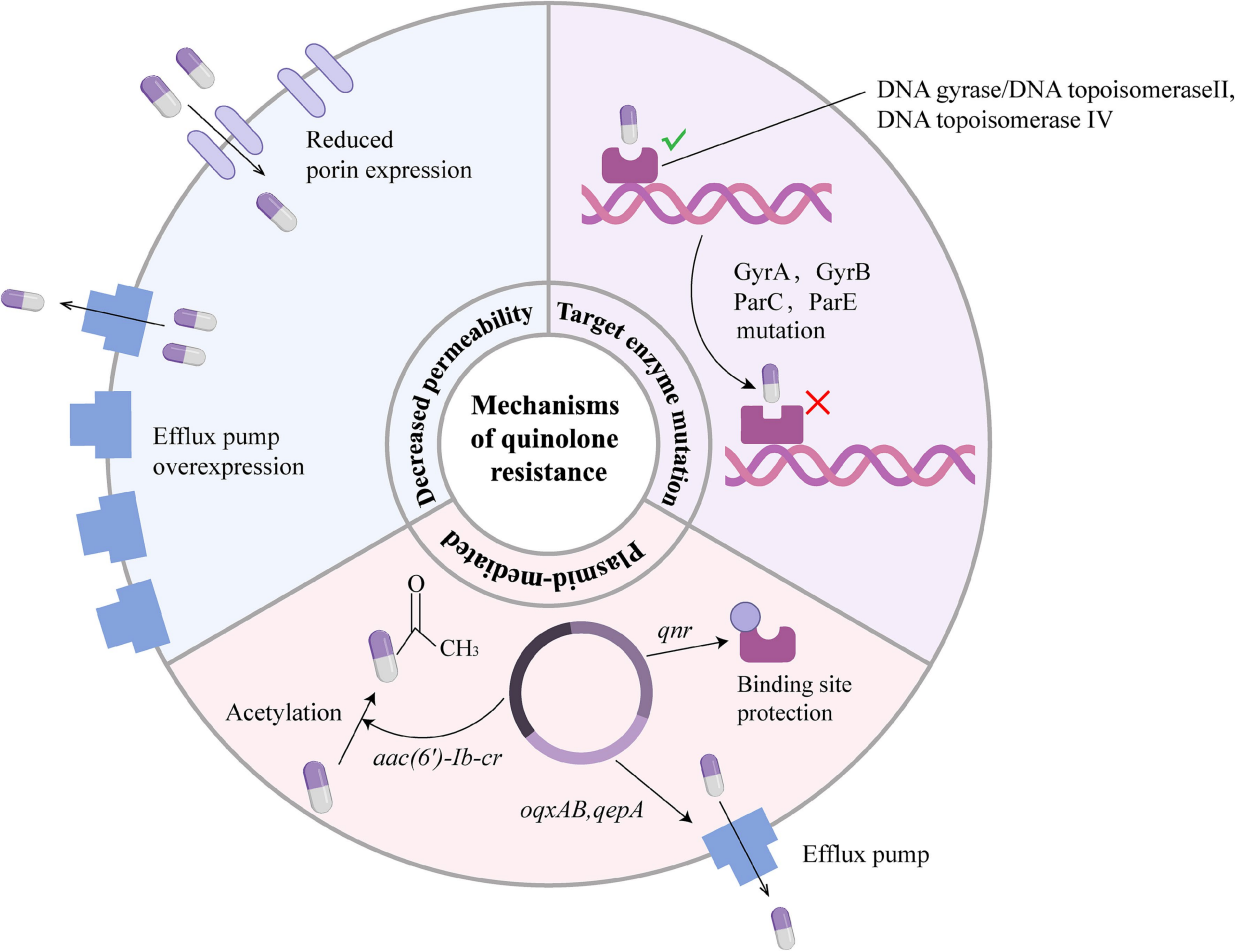
Three core resistance mechanisms of quinolone antibiotics. Bacterial resistance to quinolones primarily involves three mechanisms: ① Target enzyme mutations: Mutations in the QRDRs of DNA gyrase (GyrA/GyrB) and topoisomerase IV (ParC/ParE) prevent drug binding to the target enzymes. ② PMQR: The bacterial susceptibility to quinolones is reduced via three synergistic pathways, including protection of the target enzyme binding sites by proteins encoded by the *qnr* gene, drug acetylation modification mediated by the *aac*(6′)*-Ib-cr* gene, and drug efflux via efflux pumps encoded by the *oqxAB* and *qepA* genes. ③ Synergistic effect of membrane permeability alteration and efflux pumps: Reduced expression of outer membrane porins and overexpression of efflux pumps act synergistically to lower the effective intracellular drug concentration.

The mechanisms underlying quinolone resistance are complex and multifaceted, which can be mainly categorized into three classes (Aslam et al., 2018; Pelyuntha et al., 2022) (Figure 1). First, Target enzyme mutation: This mainly manifests as amino acid substitutions in the QRDR, involving DNA gyrase (GyrA, GyrB) and topoisomerase IV (ParC, ParE) (Laponogov et al., 2013; Hooper and Jacoby, 2015). Among these, mutations in the *gyrA* gene play a dominant role, and the accumulation of multiple-site mutations can lead to high-level drug resistance (Luo et al., 2011). Second, Acquisition of PMQR genes: The resistance conferred by these genes primarily relies on protecting binding sites of DNA gyrase via the *qnr* gene; enzymatic modification of the drug via the *aac(6′)-Ib-cr* gene; and drug expulsion from the action site via efflux pumps encoded by the *oqxAB* and *qepA* genes, all of which reduce bacterial susceptibility to quinolones (Ruiz et al., 2012; Heidary et al., 2017). PMQR genes can act synergistically with chromosomal QRDR mutations (Domínguez-Herrera et al., 2013). They not only directly increase the minimum inhibitory concentration (MIC) of quinolone, but also elevate the mutant prevention concentration and accelerate the emergence of high-level resistant strains (Kotb et al., 2019). Furthermore, horizontal transfer of plasmids enables the rapid dissemination of these resistance mechanisms. Finally, Altered drug permeability: This is predominantly observed in G^−^ bacteria (Elbehiry et al., 2025). The core mechanism is the synergistic effect of reduced expression of outer membrane porins and overexpression of efflux pumps, which decreases drug influx and accelerates drug efflux, thus significantly enhancing the level of drug resistance (Hooper and Jacoby, 2015).

In addition, unlike other bacteria, the core resistance mechanism of *Stenotrophomonas maltophilia* is target protein protection mediated by the *SmQnr* gene, which confers resistance by protecting topoisomerases. This mechanism only results in low-level resistance, with no resistance associated with mutations in the target proteins (Chang et al., 2015; Wu et al., 2019; Azimi et al., 2021).

## 3 Phage therapy

The antibacterial advantages of phages hold important clinical value. First, they have high targeting specificity without damaging the commensal flora (Huang et al., 2014; Lin et al., 2017). In addition, they are capable of self-proliferation, enabling long-lasting bactericidal effects without frequent administration (Ranveer et al., 2024). Furthermore, they can secrete depolymerases to degrade the extracellular polymeric substances (EPS) matrix of biofilms (Au et al., 2021). More importantly, they retain bactericidal activity against MDR strains and exhibit synergistic effects with antibiotics, thereby reducing antibiotic dosage and restoring drug sensitivity in resistant bacteria (Li et al., 2023; Noor et al., 2024). Finally, they have a favorable safety profile (Huang et al., 2023), with clinical data showing no significant difference in the incidence of adverse reactions compared with placebo and antibiotic groups, and no directly related serious adverse events reported to date (Ibrahim et al., 2025).

However, phage monotherapy has inherent limitations. Phages have a narrow lytic spectrum, and the emergence of phage tolerance is faster and more prevalent. Biofilm formation mediated by bacterial quorum sensing further hinders phage penetration. In addition, multiple bacterial resistance mechanisms can impair the efficacy of phages, including failed phage adsorption due to receptor mutations, specific degradation of phage genetic material by the CRISPR-Cas system, cleavage of unmethylated phage DNA by restriction-modification systems, and blockage of phage spread via abortive infection triggered by bacterial programmed cell death (Kalia et al., 2025).

## 4 Synergistic mechanisms of quinolone-based PAS

**Figure 2:**
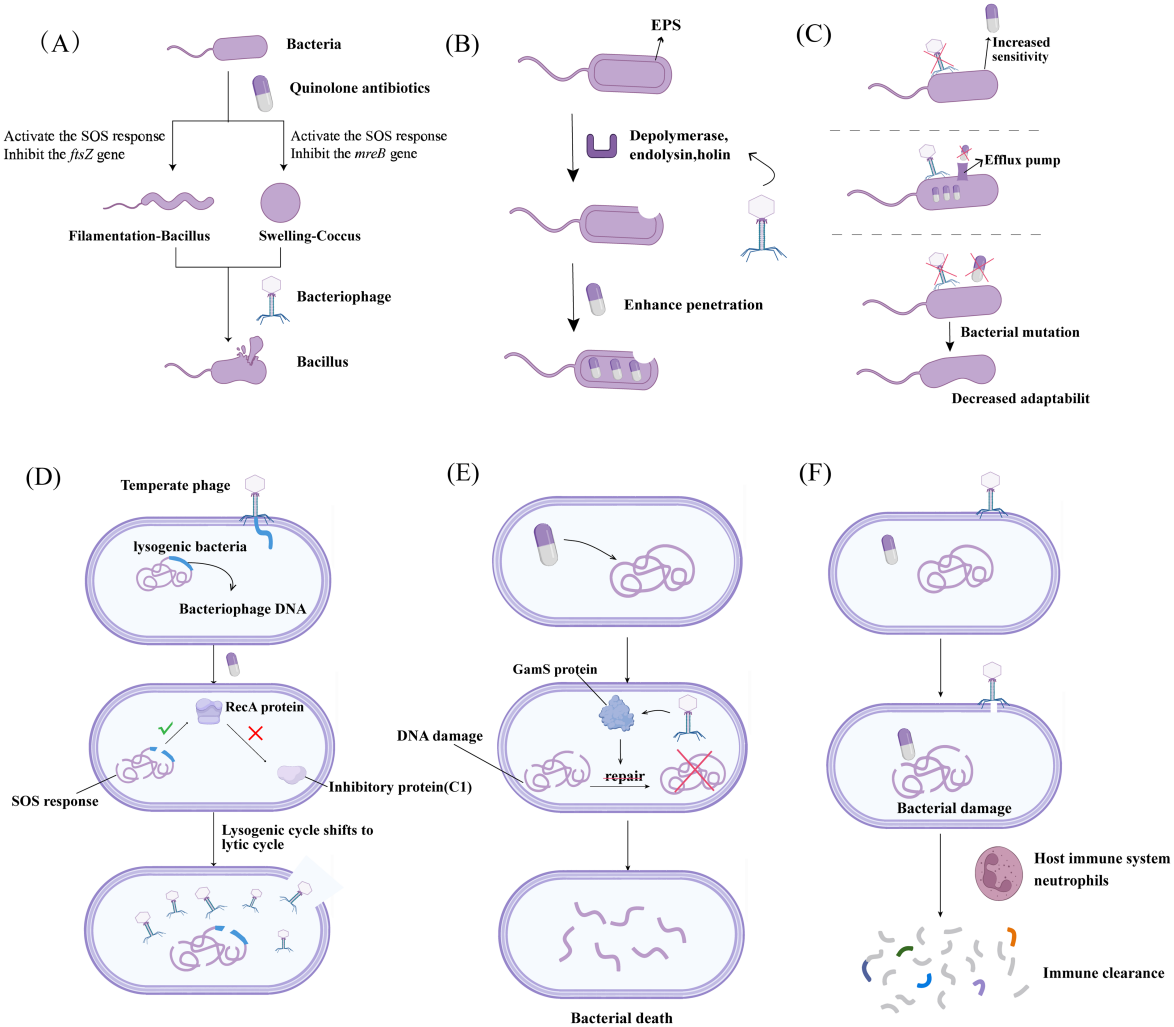
Core mechanisms of quinolone-based PAS. This figure illustrates the multidimensional mechanisms of quinolone-based PAS: **(A)** Morphological remodeling: Bacterial filamentation/swelling improves the efficiency of phage infection; **(B)** Dual selective pressure and evolutionary trade-off: Inhibits the emergence of drug-resistant mutants and impairs their bacterial fitness; **(C)** Barrier disruption: Degrades EPS and the bacterial cell wall to enhance antibiotic penetration; **(D)** Temperate phage activation: The SOS response activates lysogens and thereby drives them to enter the lytic cycle; **(E)** Synergistic action of functional proteins (exemplified by the GamS protein of phage *λ*): Proteins such as GamS inhibit DNA damage repair and amplify quinolone-induced DNA damage; **(F)** Immune synergy: The host immune system synergistically eliminates residual bacteria.

Quinolone-based PAS is a complex, multi-level and multi-target biological process, encompassing core dimensions such as bacterial physiological remodeling, molecular regulation, evolutionary trade-offs, and host immune involvement. The synergistic mechanisms between quinolone antibiotics and phages are systematically elaborated below (Figure 2).

### 4.1 Bacterial morphological remodeling

Sub-minimal inhibitory concentrations (sub-MIC) of quinolone antibiotics can induce filamentation in bacilli such as *Escherichia coli* and cell swelling in cocci such as *Staphylococcus aureus* by interfering with bacterial DNA replication and cell division processes, activating the SOS stress response (Bulssico et al., 2023), or directly inhibiting the *ftsZ* (filamentation-mediating) or *mreB* (swelling-mediating) genes (Kim et al., 2018; Bulssico et al., 2025). Notably, this effect is independent of an intact SOS system and can be consistently observed in SOS-deficient strains (Comeau et al., 2007). Reactive oxygen species (ROS) and heat stress can also induce bacterial filamentation (Bulssico et al., 2023). At sub-MIC, ciprofloxacin induces cell division arrest and filamentation in *Escherichia coli*. The expanded cell membrane surface area of these filamentous cells significantly enhances the adsorption, infection, and lysis efficiencies of bacteriophages including HK620, T4, T5, and T7, along with an increased number of intracellular viral factories, resulting in a 28–36% elevation in the burst size of phage HK620 (Bulssico et al., 2023). The core biochemical mechanism underpinning this synergistic enhancement can be elucidated by the delayed lysis hypothesis: Antibiotic-induced filamentation drives a drastic expansion of the bacterial membrane surface area, whereas the host’s synthetic capacity for holin, the key phage-encoded lytic protein, fails to increase proportionally and synchronously. The resulting insufficient holin concentration per unit membrane area necessitates a longer duration to accumulate to the threshold required for transmembrane pore formation, which in turn delays host cell lysis. This extended time window provides ample duration for intracellular phage genome replication and virion assembly, ultimately leading to a marked increase in progeny phage burst size and an amplified lytic effect (Kim et al., 2018). More critically, filamentous cells represent a hypermutable subpopulation with activated SOS response (61% of filamentous cells are SOS response-positive, versus merely 39% of normal cells). Phages can preferentially target and lyse this subpopulation, leading to a more than 5-fold reduction in ciprofloxacin-induced bacterial mutation rate (Bulssico et al., 2023). Further validation via mathematical modeling confirmed that this synergistic strategy harbors the dual advantages of rapid bactericidal activity and suppression of antibiotic resistance mutations, thus providing a novel theoretical foundation for the treatment of drug-resistant bacterial infections.

Discrepancies remain in existing studies regarding the impact of filamentation on the early stages of phage infection. Ponmalar’s study demonstrated that sub-MIC of ciprofloxacin can alter the membrane lipid dynamics of *E. coli*, which may interfere with phage adsorption (Ponmalar et al., 2022). However, a landmark study in the field published by Kim verified that filamentation does not significantly alter phage adsorption efficiency (Kim et al., 2018), suggesting that this morphological change exerts its synergistic effect primarily through modulating the lysis process during the middle and late stages of the phage life cycle. Cell swelling enhances phage efficacy by reducing the spatial occupation of bacteria and promoting phage diffusion (Bulssico et al., 2025). Importantly, morphological remodeling is not a prerequisite for the occurrence of PAS. For example, *Pseudomonas aeruginosa* does not exhibit obvious filamentation under ciprofloxacin treatment, yet significant synergistic effects can still be achieved through alternative pathways, including efflux pump regulation and biofilm degradation (Kim et al., 2018).

### 4.2 Activation of temperate phages

For lysogenic bacteria with temperate phages integrated into their genomes, quinolone antibiotics can exert synergistic bactericidal effects by modulating the life cycle of temperate phages, a mechanism defined as temperate phage-antibiotic synergy (tPAS). tPAS induces the activation of endogenous prophages within bacteria and triggers the lysis of host bacteria, with the released progeny phages exerting further bactericidal effects. This process serves as a critical supplement to the antibacterial activity of lytic phages, enables coverage of bacterial subpopulations that are insensitive to lytic phages due to lysogenic immunity, and ultimately broadens the host spectrum of the overall antibacterial strategy (Al-Anany et al., 2021). Quinolone antibiotics intercalate into the binding interface between their target enzymes (DNA gyrase and topoisomerase IV) and bacterial DNA, stabilizing the formation of the drug-enzyme-DNA ternary cleavage complex (SCC) (Pohlhaus et al., 2008). This complex directly blocks bacterial DNA replication and damage repair programs, thereby inducing DNA double-strand breaks (DSBs). The broken DNA ends are subsequently processed by the RecBCD complex to generate single-stranded DNA (ssDNA) fragments, which act as the core activation signal to trigger the bacterial SOS stress response and confer robust co-protease activity on the RecA protein (Gavric and Knezevic, 2026). Activated RecA specifically mediates the proteolytic cleavage of lysogenic repressor proteins (e.g., CI repressor) of temperate bacteriophages, disrupting the lytic-lysogenic equilibrium of the integrated prophage. This relieves the transcriptional repression of phage lytic genes, forcing the prophage to excise from the host bacterial genome and switch to the lytic cycle, which ultimately drives the lysis and killing of the host bacteria (Al-Anany et al., 2024; Fatima et al., 2025b). This mechanism not only eradicates established lysogens, but also prevents newly infected bacteria from entering the lysogenic state, reducing the lysogenization frequency from 75 to 5% and achieving highly efficient synergy with antibiotics (Fatima and Hynes, 2025b).

It should be clearly noted that the application of temperate phages in antibacterial therapy carries significant risks: the lysogenic state delays the bactericidal effect and impairs antibacterial efficacy (Monteiro et al., 2019); it may also introduce virulence genes, such as diphtheria toxin and Shiga toxin, into the host bacteria through lysogenic conversion, thereby enhancing bacterial pathogenicity (Waldor and Mekalanos, 1996). In addition, fluoroquinolones such as ciprofloxacin can induce P22-like prophages in multidrug-resistant *Salmonella* via SOS response activation, which efficiently transfer kanamycin resistance plasmids through generalized transduction (Bearson and Brunelle, 2015), and can also markedly promote the excision, replication and dissemination of *Staphylococcus aureus* pathogenicity islands (SaPIs), bringing a prominent risk of high-frequency transfer of virulence factors (Ubeda et al., 2005). What’s more, under complex infectious conditions, induced prophages compete with exogenous therapeutic phages via the superinfection exclusion (SIE) mechanism, rendering host bacteria resistant to therapeutic phages and weakening therapeutic efficacy (Bucher et al., 2024). Nevertheless, temperate phages are highly abundant in nature (Touchon et al., 2016) and have demonstrated unique potential in clinical therapy (Lauman and Dennis, 2023). Future systematic studies are required to explore their biological characteristics and controllable application pathways, so as to expand their utility in anti-infection and other biomedical fields (Monteiro et al., 2019). To address this, native lytic phages are the primary option for clinical applications, while customized engineering of temperate phages can be achieved via synthetic biology techniques: temperate phages are modified through targeted knockout of lysogeny-related genes and virulence genes, as well as targeted insertion of functional genes, to abrogate their lysogenic capacity and convert them into non-virulent, obligately lytic phages (Dedrick et al., 2019).

Notably, the functional role of the SOS response in quinolone-based PAS effects depends on the phage type. For lytic phages, classic studies show the PAS effect is intrinsically SOS-independent and mainly results from bacterial filamentation. For temperate phages, by contrast, the SOS response is the core driver of prophage induction: activated RecA directly cleaves the phage repressor to irreversibly trigger the lytic cycle.

### 4.3 Overcoming bacterial defensive barriers

Phages can target and disrupt the physical and biochemical defense systems of bacteria through multiple mechanisms, clearing the way for efficient penetration and robust bactericidal activity of quinolone antibiotics.

#### 4.3.1 Biofilm barrier disruption

Phages can eliminate the barriers to the efficacy of quinolone antibiotics by targeting and disrupting the physical defense systems of bacteria. The holins encoded by bacteriophages form micron-scale pores in the bacterial cell membrane, which enable soluble endolysins to translocate from the cytoplasm to the periplasmic space, where they access and degrade the peptidoglycan layer (Young, 2014). Endolysins specifically degrade the peptidoglycan of the bacterial cell wall, increase outer membrane permeability, and facilitate the efficient intracellular entry of antibiotics. The cell-wall binding domain (CBD) of endolysins may exhibit a broader binding spectrum than intact bacteriophages. Recent studies have challenged conventional understanding by revealing that the CBD of endolysins from *Staphylococcus* phages mediates cross-species binding. The binding specificity of this domain is determined by key amino acid residues, and it targets the conserved peptidoglycan structure of bacteria (the pentaglycine cross-bridge of *Staphylococcus aureus*) (Vázquez et al., 2025). This greatly restricts the ability of bacteria to develop mutational escape, while achieving both precise bactericidal activity and broad-spectrum coverage. Endolysins and holins have markedly heterogeneous antibacterial spectra, spanning from high species specificity to broad-spectrum activity against diverse G^−^ (and even G^+^) bacteria. Their fusion expression generally boosts antibacterial activity and spectrum width: the holin-endolysin fusion protein RL_Hlys outperforms the single endolysin RL_Lys significantly (Basit et al., 2021), and holin HolGH15 alone has a wider lytic spectrum than its cognate endolysin (Song et al., 2016). Protein engineering is the most effective strategy to overcome the bacterial outer membrane barrier and realize broad-spectrum antibacterial activity (Gutiérrez and Briers, 2021). Most bacteriophages carry polysaccharide depolymerases, which exert their functions by specifically recognizing and hydrolyzing polysaccharide structures on the bacterial surface or within biofilms, including capsular polysaccharide, extracellular polysaccharide, and lipopolysaccharide (Guo et al., 2023). These enzymes fully expose deep dormant bacteria embedded in biofilms to the action of antibacterial agents, and can increase the antibiotic concentration within biofilms by 10- to 100-fold (Glonti et al., 2010; Chang et al., 2019). Moreover, certain phages can metabolically reactivate dormant cells, thereby overcoming their intrinsic tolerance to quinolones, which target active DNA replication (Kim, 2025). It is well established that biofilms are associated with approximately 80% of human bacterial infections (Yasir et al., 2020), and represent one of the most important drivers of microbial tolerance to antibacterial agents (Flemming et al., 2016). Studies have shown that fluoroquinolones (ciprofloxacin, levofloxacin, gatifloxacin, etc.) penetrate biofilms more effectively than tobramycin and other aminoglycoside antibiotics (Chang et al., 2019; Issa et al., 2019), among which ciprofloxacin is the most widely studied. Phage-fluoroquinolone combination therapy further improves anti-biofilm effects: phage PEV20 plus ciprofloxacin outperforms monotherapy in eradicating biofilms from clinical *Pseudomonas aeruginosa* isolates (Chang et al., 2019), while phage FRZ284 combined with levofloxacin markedly reduces biofilm biomass of drug-resistant *Klebsiella pneumoniae* (Krivulia et al., 2024). This anti-biofilm effect is applicable not only to monospecies biofilms, but also to mixed-species biofilms formed by *Staphylococcus aureus* and *Pseudomonas aeruginosa* (Tkhilaishvili et al., 2020; Wang Z. et al., 2024), as well as *Pseudomonas aeruginosa* and *Candida albicans* (Roszak et al., 2022), where its efficacy is significantly superior to that of monotherapy, demonstrating excellent adaptability to complex clinical infection scenarios. For mixed-species biofilms, the core mechanism of the synergistic effect is mechanical disruption: lysis of a single species degrades the biofilm matrix, exposing other coexisting species to the killing effects of antibiotics and immune effectors. Secondary biochemical effects, such as altered quorum sensing and reduced extracellular polymer production in surviving bacteria, also contribute to the synergistic effect.

#### 4.3.2 Phage-encoded functional proteins

Functional proteins encoded by certain phages can directly potentiate the bactericidal activity of quinolone antibiotics through molecular mimicry or inhibition of bacterial DNA repair pathways. For instance, the Ref protein of phage P1 exerts synergistic enhancement effects via three-pronged mechanisms: the N-terminal domain binds to bacterial DNA and induces the SOS response; the C-terminal nuclease domain specifically cleaves RecA-bound DNA to exacerbate genome degradation; and the full-length protein inhibits bacterial cell division, preventing bacteria from escaping bactericidal pressure. Among these, the N-terminal domain of the Ref protein, enriched in positive charges and structurally disordered, anchors the protein to DNA through nonspecific binding to both single- and double-stranded DNA. Its endonuclease activity, strictly dependent on RecA, specifically cleaves DNA at RecA nucleoprotein filaments, thereby generating more extensive double-strand breaks, amplifying the bacterial SOS response, and potentiating the bactericidal efficacy of quinolone antibiotics (Ronayne et al., 2016). The nuclease activity of this protein exhibits strict RecA dependence: it only targets damage sites to exert precise bactericidal effects following quinolone-induced DNA damage and SOS response activation (Gruber et al., 2015). It can utilize RecA from different bacterial species as a cofactor to achieve cross-species synergistic effects, reducing bacterial viability by more than 2 log units (Ronayne et al., 2016). However, there are two major potential risks: in the subpopulation of incompletely eradicated bacteria, Ref-induced DSBs may trigger gene mutations and genomic rearrangements via error-prone repair, accelerating the evolution of antibiotic resistance; and severe DNA damage can potently activate the SOS response, which may reactivate endogenous prophages in the bacterial genome and cause adverse consequences such as horizontal transfer of virulence or antibiotic resistance genes.

The GamS protein of phage *λ* mimics double-stranded DNA (dsDNA) in the form of a dimer, competitively binds to RecBCD, the key enzyme mediating bacterial dsDNA break repair, and suppresses dsDNA break repair via molecular mimicry and steric hindrance. This not only enhances the bactericidal effect and reverses quinolone resistance (Wilkinson et al., 2016), but also blocks DSB repair, the upstream trigger of the SOS response, thereby indirectly suppressing SOS-mediated hypermutation and the evolution of antibiotic resistance (Revitt-Mills et al., 2022). RecBCD serves as the core immune hub for bacterial anti-phage infection, and the evolutionary arms race between phages and bacteria around this target exhibits a marked signature of convergent evolution. Studies by Wilkinson et al. confirmed that multiple phage-encoded proteins can target and modulate RecBCD function via distinct mechanisms: GamS inhibits RecBCD activity to protect its own phage genome, while gp5.9 blocks RecBCD function through substrate mimicry, and Abc2 does so by binding to the Chi recognition domain of the RecC subunit (Wilkinson et al., 2022). In response to these phage antagonistic strategies, bacteria have evolved a multi-layered synergistic defense system: they upregulate the expression of key subunits of the RecBCD complex to attenuate the inhibitory effect of GamS via a “titration” effect (Marsić et al., 1993); through the widely distributed Gabija defense system, linear phage genomes fail to undergo circularization after RecBCD is inhibited by proteins such as GamS, and are thus recognized as exogenous genetic material and eliminated by the Gabija system (Hong et al., 2025); the membrane-associated Kiwa system acts synergistically with RecBCD, where GamS can only inhibit either the RecBCD complex or the Kiwa system at a time, allowing bacteria to restrict GamS activity via this “inhibitor partitioning” effect (Zhang Z. et al., 2025). The bidirectional evolution of counterstrategies highlights the ongoing co-evolutionary arms race between bacteria and phages. This study provides critical insights into the development of novel antibacterial therapeutic strategies. Inhibitors targeting bacterial DSB repair can resensitize drug-resistant bacteria to clinically used antibiotics and suppress the emergence of *de novo* resistance mutations. Progress has been made in the development of small-molecule RecBCD inhibitors rationally designed based on the structural basis of GamS; among these, OXF-077 inhibits the evolution of bacterial antibiotic resistance by blocking DNA repair and the SOS response (Bradbury et al., 2024). Furthermore, bacterial strains harboring the Gabija or Kiwa anti-phage defense systems exhibit significantly higher tolerance to GamS-based therapy. Accordingly, pre-treatment genomic screening of pathogenic isolates is clinically recommended, to either select eligible cases with infecting strains lacking these defense systems, or develop engineered phages capable of evading host anti-phage defenses.

### 4.4 Host-phage-antibiotic regulatory network

The quinolone-based PAS effect manifests not only as unidirectional antibacterial action of antibiotics and phages, but also as complex bidirectional regulation and dynamic feedback across the host-phage-antibiotic tripartite system.

#### 4.4.1 Impacts on host metabolic homeostasis

Phages can actively interfere with the metabolic and signaling networks of the host by expressing effector proteins, exerting direct impacts on host metabolic processes, and thereby altering bacterial susceptibility to quinolone antibiotics. A typical example is the quorum sensing-targeting protein Qst expressed by the *Pseudomonas aeruginosa* phage LUZ19. This protein directly interacts with PqsD, a key enzyme in the *Pseudomonas* quinolone signal (PQS) biosynthetic pathway, resulting in decreased levels of PQS and its precursor 2-heptyl-4(1H)-quinolone. Qst also targets the central carbon metabolic enzymes CoaC and ThiD, engages a novel non-ribosomal peptide synthetase pathway designated PA1217, and blocks cell division in *Pseudomonas aeruginosa* (Hendrix et al., 2022). This complex reprogramming of host metabolism not only affects biofilm formation and maintenance, but may also indirectly modulate the uptake of and response to quinolone antibiotics by altering the physiological state of bacteria.

#### 4.4.2 Host immune synergy

Quinolone-based PAS therapy does not merely rely on direct bactericidal effects, but rather achieves highly efficient anti-infection treatment through a synergistic mode of bacterial damage and immune clearance, forming a “phage-antibiotic-immune” ternary synergistic effect. Flow cytometry analysis revealed that the proportion of damaged bacterial cells after combination treatment was as high as 95–96%, while the proportion of dead bacterial cells was only 1.24–1.47% (Jo et al., 2016a), suggesting that the core mechanism is the damage of bacterial cell membrane. Although the damaged bacteria retain partial metabolic activity, they have completely lost their proliferative capacity and pathogenicity, making them more readily recognized and completely eliminated by host immune cells, particularly phagocytes. Roach et al. demonstrated in a murine model of acute *Pseudomonas aeruginosa* pneumonia that bacteriophage therapy was effective in immunocompetent mice but completely ineffective in neutrophil-depleted mice, indicating that synergy between neutrophils and phages is essential for successful infection clearance (Roach et al., 2017). This synergistic mode not only reduces the required dosage of antibiotics and lowers the risk of drug toxicity, but also mobilizes the host’s own immune system to eliminate residual pathogens. For example, phage BD49 can stimulate host immunity and enhance lymphocyte function while exerting its antibacterial activity (Liu et al., 2024), thus forming a complete therapeutic closed loop. Phages here serve not only as a direct bactericidal tool, but also as an “immunogenic damage inducer”. In view of individual differences in immune status, future individualized schemes should comprehensively assess the patient’s immune status (such as neutrophil function, complement and humoral immunity levels), and optimize the dosing order and therapeutic window. Our group proposes to systematically dissect this ternary synergistic mechanism from the following four dimensions: at the molecular interaction level, a quaternary co-culture system of human neutrophils, bacteria, phages and quinolones is established *in vitro*, combined with multi-technology analytical platforms to analyze phagocytosis, innate immune responses and changes in bacterial membrane immune markers; at the tissue microenvironment level, co-culture of human respiratory epithelial organoids and neutrophils is applied to mimic the infection microenvironment, to dissect the synergistic dynamics of the treatment and the immune regulatory network; at the *in vivo* validation level, immune function-stratified mouse infection models are constructed to monitor bacterial clearance, resistance mutations, inflammatory responses and pathological changes; at the evolutionary prediction level, multi-dimensional data are integrated to build a population dynamics model, combined with whole-genome sequencing (WGS) and transcriptome sequencing (RNA-seq) to dissect the evolutionary trajectories of drug-resistant mutants and their susceptibility to immune clearance.

### 4.5 Dual selective pressure and evolutionary trade-off

Quinolone-based PAS exerts dual selective pressure at the evolutionary biology level, resulting in a significantly lower probability of drug resistance mutations compared with monotherapy (Jo et al., 2016b). For instance, in the treatment of multidrug-resistant (MDR) *Pseudomonas aeruginosa* with PAM2H phage cocktail plus ciprofloxacin, distinct genotypic differences were observed: all residual isolates from the phage monotherapy group harbored mutations in phage receptor genes (e.g., *pilQ, pilB*), resulting in phage resistance; whereas isolates from the combination group showed no such receptor mutations, with PH2 and CAPH1 strains carrying a deletion of the efflux pump-encoding gene *mexX* and further enhanced antibiotic susceptibility (Engeman et al., 2021). Although bacteria may evolve drug-resistant mutants, such mutants are associated with an extremely high fitness cost (Zhao et al., 2024; Parab et al., 2025; Zhang and Ahn, 2025). For example, the infection rate of *Pseudomonas aeruginosa* strains resistant to the PP1131 phage cocktail has dropped significantly; their load on heart valves is markedly lower than that of the parental strain, and they are unable to colonize the bloodstream due to virulence defects (Oechslin et al., 2017); similarly, phage resistance-associated mutations can cause damage to key metabolic pathways, as seen in *galU* gene mutants with inhibited carbohydrate metabolism and a 30% decrease in bacterial metabolic activity. Such mutants face survival limitations in the therapeutic environment and struggle to persist, thereby effectively reducing the emergence of clinically relevant stable resistant strains.

In terms of evolutionary trade-offs, surface receptor modifications in bacteria to resist phage infection are often accompanied by marked fitness costs, such as the loss of efflux pump function or membrane structural integrity. Existing evidence indicates that this effect is more an indirect consequence of evolutionary trade-offs rather than a direct effect of antibiotics. This creates a “phage resistance-antibiotic susceptibility” trade-off effect and achieves bidirectional synergistic evolutionary regulation (Chaudhry et al., 2017; Rajaure and Adhya, 2019; Engeman et al., 2021). For example, mutations in the MexAB-OprM efflux pump of *Pseudomonas aeruginosa*, which arise to resist phages, lead to reduced efflux function and a significant restoration of sensitivity to levofloxacin (Chung et al., 2026). Its profound clinical significance lies in driving a paradigm shift in antibacterial treatment logic: from “overcoming drug resistance with novel antibiotics” to “disarming drug resistance with phages”. It restores the susceptibility of bacteria to existing agents including quinolones via evolutionary selective pressure, thereby extending the clinical lifespan of these drugs. Looking ahead, phages could be precisely matched according to the drug resistance mechanisms and receptor characteristics of pathogenic bacteria, to realize individualized combination antibacterial therapy. Conversely, mutations that arise in bacteria to tolerate quinolones, including overactivation of efflux pumps (Tarasenko et al., 2025) and alterations in cell wall synthesis pathways (Parab et al., 2025), may also indirectly enhance the adsorption efficiency of phages, even provide new infection routes for phages, and further amplify the synergistic effect.

Notably, evolutionary trade-offs are not an isolated phenomenon restricted to a single mechanistic level, but rather a unifying evolutionary logic that runs through the synergistic network of quinolone-based PAS. Under the dual selective pressure of phages and antibiotics, any unilateral adaptive mutation in bacteria may occur at the cost of fitness against the other stressor, thereby suppressing the stable evolution of multidrug resistance at the bacterial population level.

## 5 Antagonistic mechanisms of quinolone-based PAS

Although the antagonistic effects between phages and quinolone antibiotics are not the dominant interaction, they can be consistently observed under specific conditions such as high antibiotic concentrations and mismatched bacterial strain characteristics. These effects are mainly attributed to the mutual interference of their mechanisms of action, alterations in bacterial physiological status, and impairment of the phage life cycle. The specific mechanisms are elaborated below.

### 5.1 High-concentration antibiotics

Quinolone antibiotics block bacterial DNA replication and transcription by inhibiting bacterial DNA gyrase and topoisomerase IV, while phage genome replication and progeny assembly are highly dependent on the host bacterium’s nucleic acid synthesis machinery and intact metabolic system. At antibiotic concentrations several-fold above the MIC, bacterial metabolism is potently inhibited, and the synthesis of nucleic acids and proteins is comprehensively blocked. This fails to provide sufficient biosynthetic substrates for phage propagation, resulting in a prolonged latent period, a marked reduction in phage burst size, and even complete abrogation of progeny phage assembly (Martinho et al., 2024; Paranos et al., 2025b). Meanwhile, high-concentration antibiotics can drive bacteria into a dormant state to form persister cells through inducing excessive activation of the SOS response: on the one hand, the extremely low metabolic activity of persister cells is difficult to be recognized and infected by phages, directly weakening the phage lysis effect; on the other hand, the overactivated SOS response can upregulate the expression of toxin genes such as *TisB*, and its encoded product significantly reduces the SOS-dependent prophage induction efficiency by inhibiting ATP synthesis and interfering with DNA repair reactions, further aggravating the inhibition of phage lysis effect (Leinberger et al., 2024).

### 5.2 Bacterial phenotypic alterations

Under the selective pressure of quinolone antibiotics, adaptive phenotypic alterations in bacteria can indirectly trigger antagonistic effects. Overexpression of bacterial efflux pumps induced by antibiotic resistance (Hooper and Jacoby, 2015), or structural changes in the cell membrane such as lipopolysaccharide (LPS) modification (Oechslin et al., 2017) and downregulated expression of outer membrane porins (Petsong et al., 2018), may mask the specific receptors recognized by phages, thereby impeding phage adsorption and invasion. In addition, the DNA repair system activated by bacteria in response to drug-induced damage may degrade invading phage nucleic acids while repairing the bacterial genome, thus disrupting the phage replication process (Zhang H. et al., 2025).

### 5.3 Imbalanced administration strategies

Imbalances in the administration sequence and concentration ratio between phages and antibiotics represent a key inducer of antagonistic effects. If antibiotics are administered prior to phages and reach the bactericidal threshold, they rapidly kill a large number of susceptible bacteria, resulting in the lack of infectable host bacteria for phages and the failure to complete the proliferation cycle (Martinho et al., 2024). Upon infection of bacteria, certain temperate phages can indirectly enhance bacterial resistance to quinolones by upregulating host genes associated with stress tolerance, including those encoding efflux pumps and DNA repair enzymes (Fatima and Hynes, 2025b). Notably, the antagonistic effects of ciprofloxacin mostly occur in high concentration ranges that are unachievable in clinical settings (Paranos et al., 2025a), and this effect can be effectively avoided by adjusting the phage concentration and optimizing the administration sequence.

## 6 Key regulatory factors of the interaction

The interaction between phages and quinolone antibiotics is regulated by multiple factors, including drug concentration, phage characteristics, bacterial strain characteristics, and the infectious microenvironment. The core influencing factors are elaborated below.

### 6.1 Administration sequence and concentration

The concentration of quinolone is the key determinant regulating the direction of the interaction between quinolones and phages, which follows a general pattern of “synergy at low concentrations and antagonism at high concentrations” (Paranos et al., 2025b). Quinolones at sub-MIC (≤1/2 MIC) mildly suppress bacterial metabolism, induce bacterial morphological alterations, and create favorable conditions for phage proliferation without disrupting the phage life cycle, representing the optimal concentration range for synergistic effects (Kanwar et al., 2026). When drug concentrations ≥2 MIC, bacterial metabolism is potently inhibited and phage replication is impaired, which readily triggers antagonistic effects (Lopes et al., 2018). In terms of administration sequence, pre-administration of phages 6–8 h before antibiotics or simultaneous co-administration of the two agents tends to produce synergistic effects, whereas pre-administration of antibiotics prior to phages is more likely to induce antagonism (Akturk et al., 2019).

### 6.2 Phage characteristics

The type, host range, and lytic properties of phages directly determine the stability of the synergistic effect. Lytic phages, with high lytic efficiency and no interference from the lysogenic cycle, result in greater synergistic stability. In contrast, although temperate phages can be activated via the SOS response, their lytic-lysogenic switch tends to cause fluctuations in the synergistic effect, accompanied by the risk of toxin gene transfer (Necel et al., 2022). Phages with high host specificity have limited application adaptability, while broad-host-range phages have a wider scope for synergistic application. Higher lytic activity of phage-encoded lysins and greater phage burst size lead to stronger disruption of bacterial physical barriers, better antibiotic penetration efficiency, and thus a more pronounced synergistic effect (Latka and Drulis-Kawa, 2020).

### 6.3 Bacterial characteristics

The antibiotic resistance profile, physiological status, and interspecies differences of bacteria are key factors driving the heterogeneity of synergistic treatment outcomes. MDR strains are more likely to exhibit antagonism due to intrinsic resistance mechanisms such as overexpression of efflux pumps and mutations in target enzymes; in contrast, antibiotic-susceptible strains or strains with a single resistance mechanism are more likely to exhibit synergy (Jo et al., 2016a). The growth phase of bacteria also affects the interaction outcome: bacteria in the logarithmic phase are metabolically active and highly susceptible to both agents, resulting in a prominent synergistic effect; whereas bacteria in the stationary phase or embedded in biofilms are metabolically quiescent with intact barrier structures, which tends to weaken treatment efficacy and even induce antagonism (Nikolic et al., 2022). Furthermore, due to the complex outer membrane structure of G^−^ bacteria, phage-mediated disruption of this barrier yields a more pronounced enhancement of the synergistic effect (Kim et al., 2018); while synergy in G^+^ bacteria relies more heavily on phage lysin activity (Lu et al., 2021).

### 6.4 Infectious microenvironment and host factors

The physicochemical conditions of the infectious microenvironment and administration strategies significantly modulate the interaction between the two. *In vivo*, serum proteins and cations such as Na^+^ and Ca^2+^ may bind to antibiotics or phages, reducing their effective concentrations and weakening the synergistic effect. In contrast, the pH and nutritional status at local infection sites such as the skin and wounds are relatively stable, which is more conducive to the effective action of both agents (Thandar et al., 2016). The host’s innate immune function is a core determinant of therapeutic efficacy. The bactericidal effects are highly dependent on the synergy of the immune system (Jo et al., 2016a), and patients with immunocompromised status face a higher risk of treatment failure.

## 7 Clinical optimization strategies for quinolone-based PAS therapy

The clinical efficacy of quinolone-based PAS therapy is influenced by multiple factors. Optimization is required in terms of administration sequence, drug concentration, phage selection, environmental conditions, and the patient’s pathological status to mitigate the risk of antagonism and maximize synergistic effects (Table 1).

**Table 1:** Optimization strategies for phage-quinolone antibiotic synergy.

Regulatory factors	Key influencing factors and mechanisms	Clinical optimization strategies	Reference
Drug concentration and administration sequence	Concentration: Synergistic effect is achieved at sub-inhibitory antibiotic concentrations (≤1/2 MIC), while antagonistic effect occurs at high concentrations (≥2 MIC). Administration sequence: Pre-administration of phages prior to antibiotics induces synergistic effects, whereas pre-administration of antibiotics prior to phages leads to antagonistic effects.	Dose matching: The minimum effective MOI is adopted for phages, and sub-MIC is used for antibiotics. Sequential administration: Phages are administered 6–8 h in advance to create favorable conditions for antibiotic penetration.	Lopes et al. (2018), Petsong et al. (2018), Akturk et al. (2019), Henriksen et al. (2019), Alharbi et al. (2023), Wang W.-X. et al. (2024), Sahu et al. (2025), Kanwar et al. (2026), and Luo et al. (2024)
Phage Characteristics	Phage type: Lytic phages confer more stable synergistic effects. Functional properties: Phages with a broad host range, high lytic activity, and polysaccharide depolymerase-encoding capacity exert more potent synergistic effects.	Optimal phage selection: Phages with a broad host range and high lytic activity are preferentially selected. Phage cocktail therapy: Cocktail combinations achieve superior antibacterial efficacy to single phages, but avoid co-administering phages that compete for the same receptors.	Gill and Hyman (2010), Chan et al. (2013), Latka and Drulis-Kawa (2020), Necel et al. (2022), and Van Helden et al. (2025)
Bacterial Characteristics	Antibiotic resistance profile: MDR strains are prone to antagonistic effects, while antibiotic-susceptible strains are more likely to develop synergistic effects. Physiological status: Bacteria in the logarithmic phase show a prominent synergistic effect, while bacteria embedded in biofilms are prone to antagonistic effects. Interspecies difference: The synergistic effect against G + bacteria is dependent on phage lysin activity, while that against G- bacteria relies on the disruption of the outer membrane barrier.	Precision targeting: Matched phages are selected according to the antibiotic resistance profile and species of the pathogens. Biofilm disruption: Polysaccharide depolymerase-producing phages are used for preconditioning.	Jo et al. (2016a), Kim et al. (2018), Latka and Drulis-Kawa (2020), Lu et al. (2021), and Nikolic et al. (2022)
Infectious microenvironment and host factors	Microenvironmental pH: The optimal synergistic effect is achieved at neutral pH, while an acidic microenvironment weakens therapeutic efficacy .*In vivo* microenvironment: Serum proteins and cations can reduce the effective concentrations of both agents Host immunity: The therapeutic efficacy of the combination is highly dependent on the host innate immune system, with a higher risk of treatment failure in immunocompromised patients.	Administration route: Targeted administration routes, including intravenous injection, nebulized inhalation, intravesical instillation, and medicated dressings, are selected according to the infection site .Immune management: The host innate immune function should be preserved, intensified monitoring should be implemented for immunocompromised patients, and the core treatment goal is to reduce the bacterial load to an immune-controllable threshold.	Jo et al. (2016a), Thandar et al. (2016), Lin et al. (2018), Jeon and Ahn (2021), Khanal et al. (2021), Bulssico et al. (2025), Rodrigues and Silva (2025), Shamsuzzaman et al. (2025), Luong et al. (2026), and Berryhill et al. (2025)

### 7.1 Optimization of administration sequence

Accumulating evidence from multiple studies has confirmed that the regimen of phage preconditioning followed by sequential administration of quinolone achieves significantly better therapeutic efficacy than simultaneous co-administration (Akturk et al., 2019). Phage preconditioning can first degrade the extracellular polysaccharide matrix of biofilms and lyse a portion of bacteria, creating favorable conditions for subsequent antibiotic penetration, and thus exerts a prominent effect against biofilm-associated infections. Meanwhile, this regimen avoids the antagonistic effects observed with simultaneous administration, where antibiotics inhibit bacterial metabolism and interfere with phage replication (Vilas Boas et al., 2016). The specific administration interval should be optimized to match the phage lytic cycle. For example, for *Pseudomonas aeruginosa* biofilms, combining ciprofloxacin with phage preconditioning 6 h later can achieve a bacterial load reduction of approximately 6 log CFU/mL, with a marked bacterial eradication (Henriksen et al., 2019). An excessively short interval (e.g., 1 h) or simultaneous administration restricts phage proliferation and weakens the synergistic effect, whereas an overly long interval (12–18 h) tends to cause bacterial regrowth or a tendency toward drug resistance, resulting in reduced antibiotic efficacy (Wang W.-X. et al., 2024). Notably, delayed antibiotic administration 8 h after prophylactic phage administration can achieve a 100% survival rate, indicating that the early intervention window is critical. However, add-on combination therapy administered 24 h later confers no additional benefit, and may even inhibit neutrophil recovery, thus routine supplementary administration is not recommended (Wang W.-X. et al., 2024). The optimal administration window should be strictly followed in clinical practice to avoid unnecessary repeated administration. The duration of treatment varies depending on the type of infection and clinical response; given the currently limited clinical data on phage combination therapy, an individualized regimen tailored to the patient’s specific infection status is recommended.

### 7.2 Optimization of dose matching

Studies have demonstrated that quinolone at sub-MIC exerts the optimal synergistic effect in combination with phages (Shariati et al., 2023). This concentration induces bacterial filamentation to promote phage proliferation, while avoiding excessive inhibition of DNA replication caused by high-concentration antibiotics. For instance, when the ciprofloxacin concentration increases to 1 × MIC, the synergistic effect weakens and phage proliferation decreases; at 8 × MIC, the synergy is abolished and even shifts to antagonism, as high-concentration antibiotics significantly inhibit phage protein synthesis and replication, resulting in a reduction in phage titer (Thandar et al., 2016). Clinically, supra-normal high-dose administration should be avoided, and the serum drug concentration should be maintained within a sub-inhibitory or moderate therapeutic range.

The phage dosage also follows the principle of an optimal applicable range. Studies have shown that for phage ФAb4B, there is no significant difference in therapeutic efficacy within the range of 1 × 10^3^ to 1 × 10^9^ PFU per animal, and there is no need to pursue an excessively high MOI. The minimum effective MOI should be preferentially selected in clinical practice: a high MOI tends to induce rapid bacterial drug resistance, while a low MOI exerts less selective pressure, delays the emergence of resistant strains, and enables sustained bactericidal activity of phages (Sahu et al., 2025; Luo et al., 2024). In summary, the synergistic regimen combining low-titer phages and sub-MIC of antibiotics should be preferentially adopted to achieve the optimal synergistic effect and delay the development of bacterial drug resistance (Alharbi et al., 2023).

### 7.3 Optimization of phage selection and compatibility

In clinical practice, phages with a broad host range, high lytic activity and high synergy with quinolone antibiotics should be preferentially selected (Latka and Drulis-Kawa, 2020), to improve the coverage of clinical isolates and bactericidal efficacy. Phages encoding polysaccharide depolymerases have more prominent advantages, as they can efficiently degrade the biofilm matrix and create favorable conditions for antibiotic penetration. Meanwhile, phages can also be genetically modified via targeted knockout of virulence and resistance genes, or armed with high-activity depolymerases to boost biofilm penetration and disruption (Meile et al., 2022). Based on the “phage resistance-antibiotic susceptibility” phenotypic trade-off, we can administer a phage cocktail to exert multiple selective pressures. Numerous *in vitro* studies have demonstrated that phage cocktails achieve superior antibacterial efficacy to single phages and can effectively reduce the emergence of drug resistance. Therefore, phage cocktail therapy can be developed to optimize clinical therapeutic outcomes (Gill and Hyman, 2010; Chan et al., 2013). Notably, phage cocktail combinations should avoid using phages from the same genus that compete for the same receptor. For example, phages EC and 109 from the *Pbunavirus genus* both target the LPS receptor; co-administration actually accelerates resistance development, whereas the anti-biofilm activity of a two-phage combination significantly improved after excluding EC (Van Helden et al., 2025). It is recommended to adopt a combination of “phages effective against the parental bacterial strain and phages active against drug-resistant strains”, which serves as a critical guarantee for achieving highly efficient, stable combination therapy with a low risk of drug resistance. Additionally, in clinical practice, rotating phage-antibiotic combinations can be adopted, with real-time PCR and whole-genome sequencing of clinical isolates used to dynamically monitor the emergence of bacterial resistance.

### 7.4 Optimization of administration route and microenvironmental adaptation

Microenvironmental pH exerts a significant impact on the efficacy of the combination therapy. For example, phage P22 is stable at pH ≥ 4, but the synergistic effect of ciprofloxacin is abolished under acidic conditions (pH = 4), and the optimal combination efficacy is achieved only at neutral pH (pH = 7) (Jeon and Ahn, 2021). The underlying mechanisms include the destruction of phage structural integrity, downregulation of surface receptor expression on host bacteria, and reduction of the antibacterial activity of antibiotics in an acidic microenvironment.

In addition, the administration route should be precisely matched to the infection scenario to improve bioavailability and minimize the loss of drug activity. Intravenous administration is preferred for systemic infections (e.g., sepsis) to ensure rapid systemic distribution of the agents. For local infections, targeted delivery methods should be selected according to the lesion site. For instance, hydrogels or nanofiber dressings for cutaneous wound infections (Bulssico et al., 2025); nebulized inhalation for respiratory tract infections (Lin et al., 2018, 2020); intravesical instillation for urinary tract infections; enteric-coated formulations resistant to gastric acid for gastrointestinal infections (Khanal et al., 2021); and phage-coated or local irrigation therapy for biofilm-associated infections such as catheter-related infections (Shamsuzzaman et al., 2025). In clinical practice, treatment regimens should be formulated based on the characteristics of pathogens, the microenvironment of the infected site, and the patient’s pathological status to achieve individualized precision therapy.

### 7.5 Optimization of immune status and individualized therapy

The integrity of the host’s innate immune function is a core determinant of the therapeutic efficacy of quinolone-based PAS therapy, as the bactericidal effect of the antibacterial agents in this regimen is highly dependent on the synergistic action of the innate immune system. In immunocompetent hosts, there is no significant difference in therapeutic efficacy between bacteriostatic and bactericidal antibiotics combined with phages (Berryhill et al., 2025). The core goal of treatment is to reduce the bacterial load to an immune-controllable threshold, with residual pathogenic bacteria eliminated by the host immune system. This provides clear guidance for clinical medication practice: the innate immune function of patients should be preserved to avoid excessive use of immunosuppressants; for immunocompromised populations such as those with neutropenia, drug resistance monitoring should be intensified, and combination therapy should be initiated when necessary to reduce the risk of infection (Roach et al., 2017); blind pursuit of complete bacterial eradication is unnecessary, and clinical benefit can be achieved by eliminating acutely pathogenic subpopulations and resetting infectious homeostasis (Luong et al., 2026). This therapy requires rigorous screening of target populations with well-defined indications and contraindications: it is only indicated for patients with MDR/XDR bacterial infections who have failed standard antibacterial treatment or are intolerant to such therapy (Marino et al., 2025); eligible patients must simultaneously have a definitive etiological diagnosis of the causative pathogen, availability of matched lytic phages against the pathogen (Onallah et al., 2024), and an infection type falling within the recommended local infection categories (Morozova et al., 2018). Bloodstream infections and intracranial infections are not currently recommended, as phages are unable to achieve effective therapeutic concentrations in blood and cerebrospinal fluid (Nguyen et al., 2026).

## 8 Current challenges and future perspectives

Despite the considerable promise of quinolone-based PAS demonstrated in preclinical laboratory studies and clinical case reports, its clinical translation is still confronted with numerous critical bottlenecks. Concerted efforts across multiple dimensions, including basic research, pharmaceutical formulation development, clinical trials, and regulatory policies, are urgently required to realize its full value in clinical practice.

### 8.1 Current challenges

Phage-antibiotic synergy therapy exhibits both significant clinical value and multiple application risks in the prevention and control of drug-resistant bacterial infections. This therapy enhances bactericidal efficacy against drug-resistant bacteria, restores antibiotic susceptibility of resistant strains, reduces antibiotic dosage and associated adverse reactions, and synergistically eliminates bacterial biofilms. Meanwhile, it protects the commensal microbiota via the high host specificity of phages, and inhibits the evolution of bacterial antibiotic resistance (Tarasenko et al., 2025). While its core risks include: bacteria are prone to rapidly develop phage resistance, which may trigger cross-resistance and alter the bacterial antibiotic resistance profile, with an inherent trade-off between bactericidal efficiency and resistance evolution risk; there are potential risks of off-target perturbation of the host microbiota and abnormal interaction with the host immune system (Burmeister et al., 2020).

Collectively, the clinical potential and inherent risks of PAS therapy highlight that its standardized clinical translation is limited by multi-dimensional unresolved challenges and knowledge gaps, which are elaborated in the following aspects: First, at the basic research level, the molecular synergistic mechanisms of the combination therapy have not been fully elucidated. The regulatory effects of phages on bacterial efflux pumps, the molecular pathways underlying phage proliferation promotion by sub-MIC of antibiotics, and the root causes of differences in synergistic effects across different bacterial species remain to be further deciphered. The validation scope of the host range of existing phages is limited, and there is a lack of systematic evaluation of clinical isolates from different regions and with different genotypes. In addition, research on phage resistance is insufficient: the mutation rate of phage resistance, the risk of cross-resistance, and the long-term evolutionary patterns of phage resistance have not been clarified, making it difficult to establish an effective prevention and control system (Fatima and Hynes, 2025a). Most existing studies focus on the binary phage-antibiotic interaction, with critical gaps in exploring the ternary “phage-antibiotic-host immune system” framework. The interaction mechanism between phages and host innate immunity remains poorly defined, making it difficult to fully elucidate individual differences in *in vivo* antibacterial efficacy. Meanwhile, the lack of a unified, standardized detection and evaluation system for synergistic effects, along with inconsistent screening methods and dosage reporting standards across studies, results in poor comparability and reproducibility of inter-study results. Moving to the pharmaceutical technology level, as live viral preparations, phages face challenges including a low degree of standardization in large-scale production and difficulties in ensuring titer stability, while the development of co-loaded composite formulations is still in the early stage (Mattila et al., 2015). At the clinical application level, the mechanisms by which individual differences such as the patient’s immune status and the microenvironment of the infected site affect therapeutic efficacy have not been clarified. There is also a lack of systematic evaluation of the potential risk of neutralizing antibody induction by repeated administration, as well as long-term safety data (Fatima and Hynes, 2025a). Meanwhile, the lack of a standardized clinical translation pathway, with most studies confined to *in vitro* screening or animal experiments and no systematic clinical translation verification workflow established, hinders the accumulation of evidence-based medical evidence for this therapy. Finally, at the regulatory policy level, phage-quinolone combination therapy has the dual attributes of a live biologic product and a chemical antibacterial agent, and therefore cannot be fully governed by the review and approval logic for traditional single-agent pharmaceuticals (Fuerst-Wilmes et al., 2025). To date, there are no specific regulatory guidelines, detailed risk control rules for combination use, or cross-regional mutual recognition standards for this class of combination therapies worldwide. In most countries, phage therapy is only administered in the form of compassionate use or individualized treatment. For example, some European countries represented by Belgium allow personalized preparation of phages as magistral preparations (Verbeken and Pirnay, 2022), while the U. S. FDA mandates review and approval via the Investigational New Drug (IND) or Biologics License Application (BLA) pathway (Yang et al., 2023). The fragmentation of global regulatory frameworks further exacerbates the compliance barriers to clinical translation, creating a vicious cycle between regulatory gaps and insufficient accumulation of high-level evidence-based medical evidence. Furthermore, the patentability of naturally occurring wild-type phages is highly challenged under global legal frameworks, especially for native sequences and unmodified phage compositions, which directly dampens the investment willingness of big pharma in this field (König, 2025).

### 8.2 Future research directions

To promote the standardized clinical translation of the combination therapy, concerted breakthroughs across multiple dimensions are required in the future. First, at the mechanistic research level, in-depth elucidation of the core molecular mechanisms of the combination therapy is needed to lay a theoretical foundation for the precision optimization of combination regimens and the development of phage-encoded synergistic effector protein-based pharmaceuticals. Meanwhile, we should expand the dimensions of therapeutic strategies, explore the ternary synergistic interaction of the “phage-antibiotic-host immune system”, and systematically elucidate the regulatory patterns of host immune status on the *in vivo* efficacy of the combination therapy. Next, at the formulation and regimen optimization level, we should establish a standardized methodological system, construct a three-tier detection system for synergistic effects featuring “checkerboard assay primary screening, time-kill curve validation, and dynamic monitoring.” (Supina and Dennis, 2025), unify the dosage reporting standards for phages (dual expression of PFU/mL and MOI) and antibiotics (μg/mL and multiples of MIC) (Fatima and Hynes, 2025a), and address the key pain point of insufficient comparability of results across different studies in the field. And standardized phage production processes should be established, and novel dosage forms tailored to different infection scenarios should be developed. Lytic phages with high efficacy at low MOI and robust biofilm penetration capacity should be screened to construct phage cocktail regimens targeting distinct bacterial receptors, which are further matched with quinolone antibiotics with prominent synergistic effects, to maximize the antibacterial efficacy of the combination. Furthermore, at the clinical research level, we will establish and follow the stepwise translational pathway progressing from in vitro screening, to preclinical animal models, followed by compassionate use, and culminating in a randomized controlled trial (Rodrigues and Silva, 2025), to systematically validate the in vivo efficacy and safety of the combination therapy. And multicenter randomized controlled trials (RCTs) should be conducted focusing on key indications, including MDR *Pseudomonas aeruginosa* infections, mixed-species biofilm infections, and re-sensitization therapy for fluoroquinolone-resistant bacteria. These trials should define the optimal indications, administration sequence, dosage, and treatment course, with the incorporation of immune status monitoring. The clinical monitoring protocol can be developed with reference to the standardized framework of the KIDNAP protocol, combined with WGS-based dynamic monitoring and a high-throughput screening platform, to construct a full end-to-end closed-loop workflow covering pre-treatment analysis of drug resistance mechanisms, followed by real-time intra-treatment monitoring, and ending with long-term post-treatment follow-up. Among them, qPCR enables rapid assessment of dynamic changes in bacterial load, while WGS allows in-depth analysis of the genotypic characteristics of drug-resistant mutants and their impacts on bacterial virulence and fitness (Najjar et al., 2026). Finally, at the regulatory and standardization level, future efforts should prioritize international regulatory collaboration to build a harmonized framework tailored to its dual live biologic-chemical attributes, unify core quality standards and cross-regional clinical data recognition mechanisms to resolve global regulatory fragmentation; conduct standardized international multicenter randomized controlled trials to accumulate high-level clinical evidence and break the vicious cycle of regulatory gaps and insufficient evidence accumulation; optimize phage-related intellectual property rules and innovate clinical translation models to boost investment willingness from the pharmaceutical industry; and advance GMP-compliant standardized phage production and rapid point-of-care susceptibility testing technologies, ultimately facilitating the full clinical translation of this promising regimen to combat the global public health crisis of antimicrobial resistance.
